# Bayesian hierarchical spatial count modeling of taxi speeding events based on GPS trajectory data

**DOI:** 10.1371/journal.pone.0241860

**Published:** 2020-11-13

**Authors:** Haiyue Liu, Chuanyun Fu, Chaozhe Jiang, Yue Zhou, Chengyuan Mao, Jining Zhang

**Affiliations:** 1 School of Transportation and Logistics, Southwest Jiaotong University, Chengdu, China; 2 National United Engineering Laboratory of Integrated and Intelligent Transportation, Southwest Jiaotong University, Chengdu, China; 3 National Engineering Laboratory of Integrated Transportation Big Data Application Technology, Southwest Jiaotong University, Chengdu, China; 4 College of Engineering, Zhejiang Normal University, Zhejiang, China; Tongii University, CHINA

## Abstract

Speeding behavior, especially serious speeding, is more common in taxi driver than other driving population due to their high exposure under traffic environment, which increases the risk of being involved in crashes. In order to prevent the taxi and other road users from speed-related crash, previous studies have revealed contributors of demographic and driving operation affecting taxi speeding frequency. However, researches regarding road factors, and spatial effect are typically rare. For this sake, the current study explores the contributions of 10 types of road characteristics and two kinds of spatial effects (spatial correlation and spatial heterogeneity) on taxi total speeding and serious speeding frequency. Taxi GPS trajectory data in a Chinese metropolis were used to identify speeding event. The study then established four kinds of Bayesian hierarchical count models base on Poisson and negative binominal distribution to estimate the contributor impacts, respectively. Results show that Bayesian hierarchical spatial Poisson log-linear model is optimum for fitting both total and serious speeding frequency. For the analysis, it is found that drivers are more likely to commit speeding on long multilane road with median strip, and road with non-motorized vehicle lane, bus-only lane and viaduct or road tunnel. Roads with low speed limit, and work zone are associated with increasing speeding as well. In terms of serious speeding, bus-only lane is not a contributor, while road speed camera number and one-way organization are significantly positive to the speeding frequency. Furthermore, it reveals that two spatial effects significantly increase the occurrence of speeding events; the impact of spatial heterogeneity is more critical.

## Introduction

Speeding is referred as one of the most key factors associates with serious traffic crashes around the world [1,2], causing serious losses in recent years. According to the statistics of the United States in 2017, nearly one third of fatal crashes were caused by speeding [3]. An Australian report showed that fatality of speeding in New South Wales were accounted for 40% among the total number of deaths in 2014 [4]. This issue is also prevalent in China. Speeding has been listed as the sixth contributor of traffic crashes in Chinese metropolises, with 2.1% of crashes caused by speeding events in 2016 [5].

It is noted that the speeding behavior is quite common in urban commercial vehicle drivers such as taxi population. One study found about 64.2% taxi drivers who responded the survey are involved in speed violation [6]. Given the unobserved samples, this ratio may surely be higher in practice. The finding was reinforced by another study in Beijing as well [7].

Because of the serious driving risk produced by speeding, it is urgent to control the frequent and high-risk speeding issue of taxi population primarily. To do so, number of studies explored the influence factors of taxi speeding.

The existing researches regarding influence factors of taxi speeding are mainly focus on driver’s socio-demographic factors (such as sex, age, and education) and operation characteristic (such as driving distance). Tseng [8] found that gender is insignificant with respect to speeding behavior. Yeh et al [9] and Tseng [8] explored the impact of age, revealing young drivers are more likely to exceed the speed limit than older drivers, while the study of Newnam et al [10] showed that no age deference is found in the occurrence of speeding behavior. Meanwhile, some mutual contradiction conclusions are also presented in respect of education. Studies indicated that education level of driver is not related to their speeding behavior [8,10], while another study found that lower educated female taxi drivers engage in more speeding violation [9].

Compared to the socio-demographic factors, the result of driving distance is more consistent. Driver associated with long-distance trips were believed to commit more speeding [6,8,11].

In addition to the factors above, road characteristic is seldom considered, though it has been proved to significantly impact the taxi speeding behaviors. Road factors, which determine the conditions of vehicle operation, are critical to stimulate or restrain the behavior of drivers. For example, existing studies have investigated the influences of road speed limit and segment length, suggesting that the drivers are intend to exceed speed limit on road with lower speed limits [6] and on segment with lengths from 1.0 to 1.5 km [11]. However, in terms of the speeding contributor, the impacts of other road factors such as road design and organization are unknown.

Generally, the scarcity and diversity results of taxi speeding researches can be explained as the lack of reliable data source. Unlike crash data, most speeding behavior may not be detected and recorded due to the technique limitation, causing the inadequacy of sample which leads to contradictory findings occasionally. In previous studies, taxi speeding data are typically collected by questionnaire [7–10] or practical observations such as radar gun or speed camera [6,12]. The survey approach of questionnaire may involve obviously self-perception bias, affected by not only participant attitude but also memories. The radar gun or speed camera (known as automated enforcement) can only capture speeding data in the vicinity of the enforcement site, rather than the whole trace of the vehicle. Meanwhile, the reliability of those data is easily impacted by environment and traffic condition. To overcome the data bias, several researches used GPS trajectory data to examine taxi speeding events [11,13]. These studies showed the extensive usage of GPS is able to capture a large amount of vehicle trajectory data. Researchers therefore can identify the driving behaviors on each road by processing driving features such as speed, time, position, etc. Hence, it is possible for exploring taxi speeding behavior effectively on different kinds of road conditions based on taxi GPS trajectory.

Additionally, despite the common factors used in taxi speeding researches, the absence of spatial effect may be another significant way to interpret the diversity results of taxi speeding behavior. Recent studies have confirmed that spatial effect play a critical role in affecting some driving behaviors. For example, the studies used Bayesian model with spatial effect to estimate the traffic crash with errors caused by spatial component, raising the fitness and explanatory ability of the crash contributor [14–17]. As to speeding research, an latest study held by Fu et al [13] have identified the existence of spatial autocorrelation of taxi speeding events with different severity level. However, the introduction of spatial effect is unseen in previous researches [18–20]. Thus, spatial effect is supposed to be considered for the purpose of exploring the taxi speeding precisely.

The main purpose of this study is to explore the influence of road factors on taxi speeding by introducing spatial effect. The remainder of this paper is organized as follows. Frist of all, taxi speeding events were extracted form GPS trajectory data, which labeled as total speeding events. Serious speeding events were also identified by filtering the total speeding events then. Second, count models based on Poisson and negative-binominal distribution were built, including Bayesian hierarchical structure with log-linear form to the expected value terms. The spatial effect (spatial correlation and heterogeneity) was added to the corresponding models. Therefore, the study established four models, including Bayesian hierarchical Poisson log-linear model (BHPLM), Bayesian hierarchical negative binomial log-linear model (BHNBLM), Bayesian hierarchical spatial Poisson log-linear model (BHSPLM), and Bayesian hierarchical spatial negative binomial log-linear model (BHSNBLM). All the four models were adopted to fit the total speeding and serious speeding event, respectively. To this end, the results and interpretations of factor estimation were discussed.

## Materials and methodology

### Data collection and processing

Data used in this study were collected in Chengdu, a metropolis in southwestern China. A rectangle with four edge GPS points coordinates: (104.0420, 30.65281), (104.0420, 30.72815), (104.1297, 30.65281), and (104.1297, 30.72815) are selected as study area. This area, which contains a series of major transportation attractions, produces a large number of taxi activities and includes large taxi GPS data scale as well as the known road characteristic information.

Taxi GPS trajectory data, posted speed limits and other road features within this area have been collected and measured in advance. The processes of GPS data and road information are shown as follows.

Taxi GPS dataset were collected from the taxi company in Chengdu, ranging from Nov. 1^st^ to 14^th^, 2016 (14 days). All the taxi drivers who provided this data have known the existence of GPS equipment which captured in the trajectory information during their work, and they had been informed that those data might be used in traffic research in company employment contract. Authors of this study did not access to the personally identifiable information of taxi drivers. The details of each taxi’s GPS trajectory were recorded, including date, location, longitude, latitude, company, plate number, speed, angle, vehicle type, passenger occupancy, and record time. In order to fix the position error of raw GPS data and precisely identify speeding behavior, a map matching process was imposed to match GPS trajectory point to its corresponding road segment. To be specific, the grid-based searching algorithm was implemented to find the road segments within grid of each GPS record whose length set as 100 m. The coordinates of roads central line were measured manually. GPS trajectory points were matched to the nearest road central line using an adjusted Euclidean distance. Moreover, the distance between GPS record location and intersection was calculated. Since the uncertainty of intersection speed limits., the GPS trajectory points which located within the range of 30 m around an intersection were suspended to be analyzed. Finally, the current study also implemented an anomaly detection algorithm to eliminate the abnormal driving activities such as U-turn and wrong-way driving.

The speeding event can be identified after the map matching of GPS trajectory data. To do so, the study compared the average driving speed between two adjacent GPS points with the corresponding road’s posted speed limit. The GPS points pairwise whose average travel speed exceeds the posted speed limit is identified as speeding points pairwise. Moreover, serious speeding is also explored in this study. According to the traffic law of China, speeding points pairwise whose average speed exceeds 20% over the posted speed limit is identified as serious speeding.

The average driving speed between two adjacent GPS points is calculated as:

vi,i+1=ΔDi,i+1ΔTi,i+1 (1)

where, *i* and *i*+1 are two adjacent GPS points; Δ*D*_*i*,*i*+1_ is the vehicle driving distance between GPS point *i* and GPS point *i* +1; Δ*T*_*i*,*i*+1_ is the travel time between the GPS point *i* and GPS point *i* +1; *V*_*i*,*i*+1_ is the average speed between the GPS point *i* and GPS point *i*+1.

Note that the adjacent speeding pairwise identified above can be merged into one complete speeding pairwise. To be specific, if the average speeds of adjacent points pairwise (*i*, *i*+1), (*i*+1, *i*+2) and (*i*+2, *i*+3) all exceed the speed limit, the three speeding point pairwise can be combined as speeding pairwise (*i*, *i*+3), and thus be regarded as a complete speeding event; if only pairwise (*i*+1, *i*+2) exceeds the speed limits, this isolated speeding point pairwise thus can be identified as a complete speeding event as well. The combination of adjacent speeding events is due to the GPS device frequency limitation (10s), which may divide a whole speeding event into several successive speeding record in dataset.

After identification, 650,363 total speeding events and 201,924 serious speeding events were identified in the study area.

Besides the process of speeding behavior data, road factors data on main road within study area were obtained from both official information and practical observation, which contain 10 kinds of road characteristics. Those road factors can be classified as road geometry factors and road organization factors. Road geometry factors are determined by design and construction, including number of lanes, road cross sections, the existence of viaduct or road tunnel and length of road segment. Specifically, the road cross section is associated with lane divider (e.g., median strip) which is used to convey the condition of opposite traffic flow on road surface. Viaduct is the elevated passageway mounted above the road, providing straight passageway to vehicles passing the busy area or arterial roads quickly. The road tunnel in Chengdu is typically built in busy intersection or central business district (CBD), offering a faster, uninterrupted road for vehicles to pass the intersection or transfer to another driving direction. Road organization factors refer to the road characteristic used to control vehicle driving rules, which includes speed limit, number of speed camera, the existence of one-way road, non-motorized vehicle lane, bus-only lane, and work zone.

Collinearity diagnostic has analyzed in this study and the VIF values of all the factor are far less than 10, indicating there is no collinearity existed in road factors. All factors of road segment in dataset is shown in Table 1.

**Table 1:** Descriptive statistics of collected variables.

Variable	Variable description	Mean	S.d.	Min	Max	VIF
Speed limit indicator	1 if it is equal to or less than 40 (km/h), 0 otherwise	0.21	0.41	0	1	1.384
Number of speed cameras	The number of speed cameras on the road segment	0.625	0.792	0	4	2.417
Number of lanes indicator	1 if it is equal to or less than 4, 0 otherwise	0.231	0.422	0	1	1.246
Road cross section indicator	1 if the road cross section without dividers, 0 otherwise.	0.555	0.623	0	1	1.373
One-way road indicator	1 if there exists one-way road, 0 otherwise	0.043	0.203	0	1	1.857
Non-motorized vehicle lane indicator	1 if there exists non-motorized vehicle lane, 0 otherwise	0.891	0.312	0	1	2.492
Bus-only lane indicator	1 if there exists bus-only lane, 0 otherwise	0.625	0.484	0	1	1.669
Viaduct or road tunnel indicator	1 if there exists viaduct or road tunnel, 0 otherwise	0.090	0.286	0	1	2.676
Work zone indicator	1 if there exists work zone, 0 otherwise	0.102	0.302	0	1	1.454
Length of road segment	Continuous variable (m)	162.412	88.586	4.386	489.948	2.304

Roads in study area were divided by the lane-setting and road cross sections for road factors. For instance, if a road consists of one segment with 6 lanes and another segment with 8 lanes, the road is therefore divided into two road segments. As a result, 256 road segments are contained in this study.

### Methodology

In this study, the count model based on Bayesian hierarchical architecture was developed to explore and analyze the influence factors on speeding (total and serious) events. Models with Poisson and negative-binominal distributions were considered. Spatial effect was accounted for the purpose of explaining the geographic contribution of variables. Therefore, the study established Bayesian hierarchical ordinary count models (BHPLM, BHNBLM) and Bayesian hierarchical spatial count models (BHSPLM, BHSNBLM). Three-hierarchical structure was employed in each model, which contains data layer, process layer and parameter layer. The study set the prior distributions and initial values of each hyper-parameter in advance. The details of each model are presented in follow.

**BHPLM.** In this model, the speeding frequencies are assumed to be Poisson distributed, which is the data layer of the hierarchy architecture

$$Y_{s}∼poisson(\lambda_{s})$$ (2)

where, *Y*_*s*_ is the observational value of speeding (total or serious) frequency on road segment *s*; *λ*_*s*_ is the corresponding Poisson rate (or known as expected value).

Then, the process layer of Bayesian hierarchical architecture is presented, which elected Poisson rate to be logarithm distributed, establishing a log-linear equation with factors and intercept [15].

$$log(\lambda_{s})=\sum_{k=1}^{m}X_{ks}\beta_{k}+a_{0}$$ (3)

where, *X*_*k*s_ is the *k*th influence factor on road segment *s*; *β*_*k*_ is the regression coefficient corresponding to *k*th factor; *a*_0_ is the intercept term; *m* is the number of different influence factors.

Finally, appropriate priors are assumed in the parameter level of Bayesian hierarchical architecture. In this layer, *β*_*k*_ is assumed to be normally distributed with a mean of 0 and a variance of 10000, *β*_*k*_~*N*(0,10000); *a*_0_ is assigned a normal prior *a*_0_~*N*(0,10000).

**BHSPLM.** In order to establish the models with spatial effect, spatial components including spatial correlation and heterogeneity were added into the log-linear structure in BHPLM. Thus, BHSPLM is assumed as

$$Y_{s}∼poisson(\lambda_{s})$$ (4)

$$log(\lambda_{s})=a_{0}+\sum_{k=1}^{m}X_{ks}\beta_{k}+\mu_{s}+v_{s}$$ (5)

where, *μ*_*s*_ and *v*_*s*_ are spatial correlation effect and spatial heterogeneity effect on road segment *s*, respectively. The prior distribution assumptions of *β*_*k*_ and *a*_0_ are equal to the BHPLM: *β*_*k*_~*N*(0,10000); *a*_0_~*N*(0,10000).

Spatial correlation effect refers to as a phenomenon that sample values are interactive due to their continuity of geography. For example, road in business district is likely to be associated with higher speeding frequency [13], which may cause high speeding rate on its surrounding roads. Spatial heterogeneity effect presents the discrepancy of sample value over the study area due to the geographic difference. For instance, the speeding frequency between road in business district and road in school is different; frequency of speeding on school road is usually less than road in business district.

The spatial components considered in the current study follow the Besag-York Mollie model (BYM model) [21]. Specifically, *μ*_*s*_ is assumed to intrinsic conditional autoregressive structure, while *v*_*s*_ is assumed to follow a normal distribution, as shown below:

$$\mu_{s}|\mu_{-s}∼N(\frac{\sum_{x∼s}w_{sx}\mu_{x}}{\sum_{x∼s}w_{sx}},\frac{\sigma_{\mu }{}^{2}}{\sum_{x∼s}w_{sx}})$$ (6)

$$v_{s}∼N(0,\sigma_{v}{}^{2})$$ (7)

where, *σ*_*μ*_^2^ and *σ*_*v*_^2^ are two independent variances of speeding (total or serious) events in space; *μ*_-*s*_ is the neighbors of road segments *s*, *x*~*s* refer to all the neighbors of road segment *s*, *w*_*sx*_ denotes the spatial weight matrix, which contains the spatial relationship of adjacent road between road segment *s* and road segment *x*, it can have two optional values: *w*_*sx*_ = 1 if road segments *s* and *x* are adjacent, and *w*_*sx*_ = 0 otherwise; *σ*_*μ*_^2^ and *σ*_*v*_^2^ are assigned inverse gamma priors: *σ*_*μ*_^2^~*Inverse Gamma*(0.5,0.00005), *σ*_*v*_^2^~*Inverse Gamma*(0.5,0.00005).

The study also employed spatial fraction analysis to compare the contribution of spatial correlation and spatial heterogeneity, which is given by

$$frac=\frac{\sigma_{\mu }{}^{2}}{\sigma_{\mu }{}^{2}+\sigma_{v}{}^{2}}$$ (8)

If the value of spatial fraction is close to 1, the spatial correlation effect is regarded as the main contributor in spatial structure; otherwise, the impact of spatial heterogeneity is more critical.

**BHNBLM.** Poisson distribution fits the data without over-dispersed distribution, which means mean value equals to the variance [16]. However, speeding frequency on various roads significantly differ in variance. To address this problem, the current paper introduced negative-binominal distribution as a comparison to explore the adaptability of count model, as negative-binominal distribution is commonly used in fitting over-dispersed traffic safety data. The negative-binominal distribution of speeding events in data layer can be presented as [22].

$$f\left(y_{s}|r_{s},p_{s}\right)=\frac{\Gamma \left(y_{s}+r_{s}\right)}{\Gamma \left(r_{s}\right)\Gamma \left(y_{s}+1\right)}p_{s}{}^{r_{s}}{\left(1-p_{s}\right)}^{y_{s}}$$ (9)

where, *y*_*s*_ is the frequency of speeding (total or serious) events on road segment *s*; *r*_*s*_ is the frequency of non-speeding (or non-serious) events on road segment *s*, *p*_*s*_ is the probability of *r*_*s*_ times non-speeding (or non-serious speeding) events occur when repeat *y*_*s*_+*r*_*s*_ times Bernoulli experiment until *y*_*s*_ times speeding or serious speeding events take place.

Moreover, similar with the structure of BHPLM, the study added the logarithm form in the negative-binomial model to its expected value term *λ*_s_ at the process layer. Thus, the BHNBLM can be expressed as Eqs (10) and (11), respectively.

ps=rs(λs+rs) (10)

$$log(\lambda_{s})=a_{0}+\sum_{k=1}^{m}X_{ks}\beta_{k}$$ (11)

where, *λ*_s_ is the mean predicted frequency for speeding (total or serious) events occurred on road segment *s*; *X*_*k*s_ is the *k*th influence factor vector on road segment *s*; *β*_*k*_ is the regression coefficient corresponding to *k*th factor; *a*_0_ is the intercept term; *m* is the number of influence factors.

At the parameter layer, the prior assumption of *β*_*k*_ and *a*_0_ are equal to BHPLM and BHSPLM. *r*_*s*_ is assigned a gamma prior: *r*_*s*_~*Gamma*(5,05).

**BHSNBLM.** On the basis of BHNBLM, the spatial effect including spatial correlation and heterogeneity were introduced. Thus, the study built the BHSNBLM as follows

$$f(y_{s}|r_{s},p_{s})=\frac{\Gamma (y_{s}+r_{s})}{\Gamma (r_{s})\Gamma (y_{s}+1)}p_{s}{}^{r_{s}}{\left(1-p_{s}\right)}^{y_{s}}$$ (12)

ps=rs(λs+rs) (13)

$$log(\lambda_{s})=a_{0}+\sum_{k=1}^{m}X_{ks}\beta_{k}+\mu_{s}+v_{s}$$ (14)

The priors of *β*_*k*_, *a*_0_, *r*_*s*_, *μ*_*s*_ and *v*_*s*_ are similar with the models above.

**Model estimation method.** In order to uncover the accurate impact of factors, Bayesian method is adopted to estimated parameters by calculating their posterior distributions through prior distributions. This process is known as Bayesian evaluation [23,24], which is shown in Eq (15).

f(Θ|Y)∝f(Y|Θ)π(Θ) (15)

where, *f*(Θ|Y) denotes the posterior distribution of parameters Θ; *f*(Y|Θ) represents the likelihood conditional function of parameters Θ; *π*(Θ) is the prior distribution of parameters Θ.

The prior distributions for random parameters of the four models used in the current study are assumed as follows

$$\beta_{k}∼N(a_{k},b_{k}),\;a_{0}∼N(\alpha_{0},b_{0}),\;r∼Gamma(e_{0},j_{0})$$ (16)

$$\sigma_{\mu }{}^{2}∼Inverse\;Gamma(q_{s},\rho_{s}),\;\sigma_{v}{}^{2}∼Inverse\;Gamma(x_{s},z_{s})$$ (17)

Based on the Bayesian method, the posterior joint distributions of parameters Θ in BHPLM and BHSPLM, can be derived as

$$\begin{array}{c} f(\Theta |Y)\propto f(Y|\Theta )\pi (\Theta )=\prod_{s=1}^{n}f\left(y_{s}|\beta_{k},a_{0}\right)\times \prod_{k=1}^{m}N\left(\beta_{k}|a_{k},b_{k}\right)\\ \times N\left(a_{0}|\alpha_{0},b_{0}\right) \end{array}$$ (18)

$$\begin{array}{l} f(\Theta |Y)\propto f(Y|\Theta )\pi (\Theta )=\prod_{s=1}^{n}f\left(y_{s}|\beta_{k},a_{0},u_{s},v_{s}\right)\times \prod_{k=1}^{m}N\left(\beta_{k}|a_{k},b_{k}\right)\\ \;\times N\left(a_{0}|\alpha_{0},b_{0}\right)\times \prod_{s=1}^{n}N\left(\mu_{s}|c_{0},\sigma_{u}{}^{2}\right)\\ \;\times \prod_{s=1}^{n}N\left(v_{s}|d_{0},\sigma_{v}{}^{2}\right)\times IG(\sigma_{\mu }{}^{2}|q_{0},\rho_{0})\\ \;\times IG(\sigma_{v}{}^{2}|x_{0},z_{0}) \end{array}$$ (19)

And the posterior joint distribution of Θ for both BHNBM and BHSNBM are derived as

$$\begin{array}{l} f(\Theta |Y)\propto f(Y|\Theta )\pi (\Theta )=\prod_{s=1}^{n}f\left(y_{s}|\beta_{k},a_{0},r_{s}\right)\times \prod_{k=1}^{m}N\left(\beta_{k}|a_{k},b_{k}\right)\\ \;\times N\left(a_{0}|\alpha_{0},b_{0}\right)\times \prod_{s=1}^{n}N\left(r_{s}|e_{0},j_{0}\right) \end{array}$$ (20)

$$\begin{array}{l} f(\Theta |Y)\propto f(Y|\Theta )\pi (\Theta )=\prod_{s=1}^{n}f\left(y_{s}|\beta_{k},a_{0},u_{s},v_{s},r_{s}\right)\times \prod_{k=1}^{m}N\left(\beta_{k}|a_{k},b_{k}\right)\\ \;\times N\left(a_{0}|\alpha_{0},b_{0}\right)\times \prod_{s=1}^{n}N\left(\mu_{s}|c_{0},\sigma_{\mu }{}^{2}\right)\\ \;\times \prod_{s=1}^{n}N\left(v_{s}|d_{0},\sigma_{v}{}^{2}\right)\times IG(\sigma_{\mu }{}^{2}|q_{0},\rho_{0})\\ \;\times IG(\sigma_{v}{}^{2}|x_{0},z_{0})\times \prod_{s=1}^{n}N(r_{s}|e_{0},j_{0}) \end{array}$$ (21)

In each model, the prior distributions of hyper parameters shown in Eqs (22) and (23) are set as

$$a_{k}=\alpha_{0}=c_{0}=d_{0}=0,\;b_{k}=b_{0}=10000,\;q_{0}=x_{0}=0.5$$ (22)

$$\rho_{0}=z_{0}=0.00005,\;e_{0}=5,\;j_{0}=0.5$$ (23)

**Model comparison.** Deviance Information Criteria (DIC) is introduced to comprehensively compare the fitting ability of four models above. It is commonly applicable to the model with uncertain values of parameters such as Bayesian hierarchical model calculated by Markov Chain Monte Carlo (MCMC). DIC is defined as follows [25,26]:

$$DIC=\bar{D}+p_{d}=D(\bar{\theta })+2p_{d}$$ (24)

where, $D(\bar{\theta })$ is the deviation at the posterior mean; $\bar{D}$ is the posterior mean of $D(\bar{\theta })$; *p*_*d*_ is the number of effective parameters of the model.

Both the fitting state (represented by $\bar{D}$) and the complexity of the model (represented by *p*_*d*_) can be simultaneously examined by DIC. For the evaluation result, the smaller the DIC value is, the better performance the model has. If the DIC values of the two models differ within 5, the fitting of the two models are roughly the same; otherwise, the model with a smaller DIC value is better [27].

## Results and discussion

**Fig 1:**
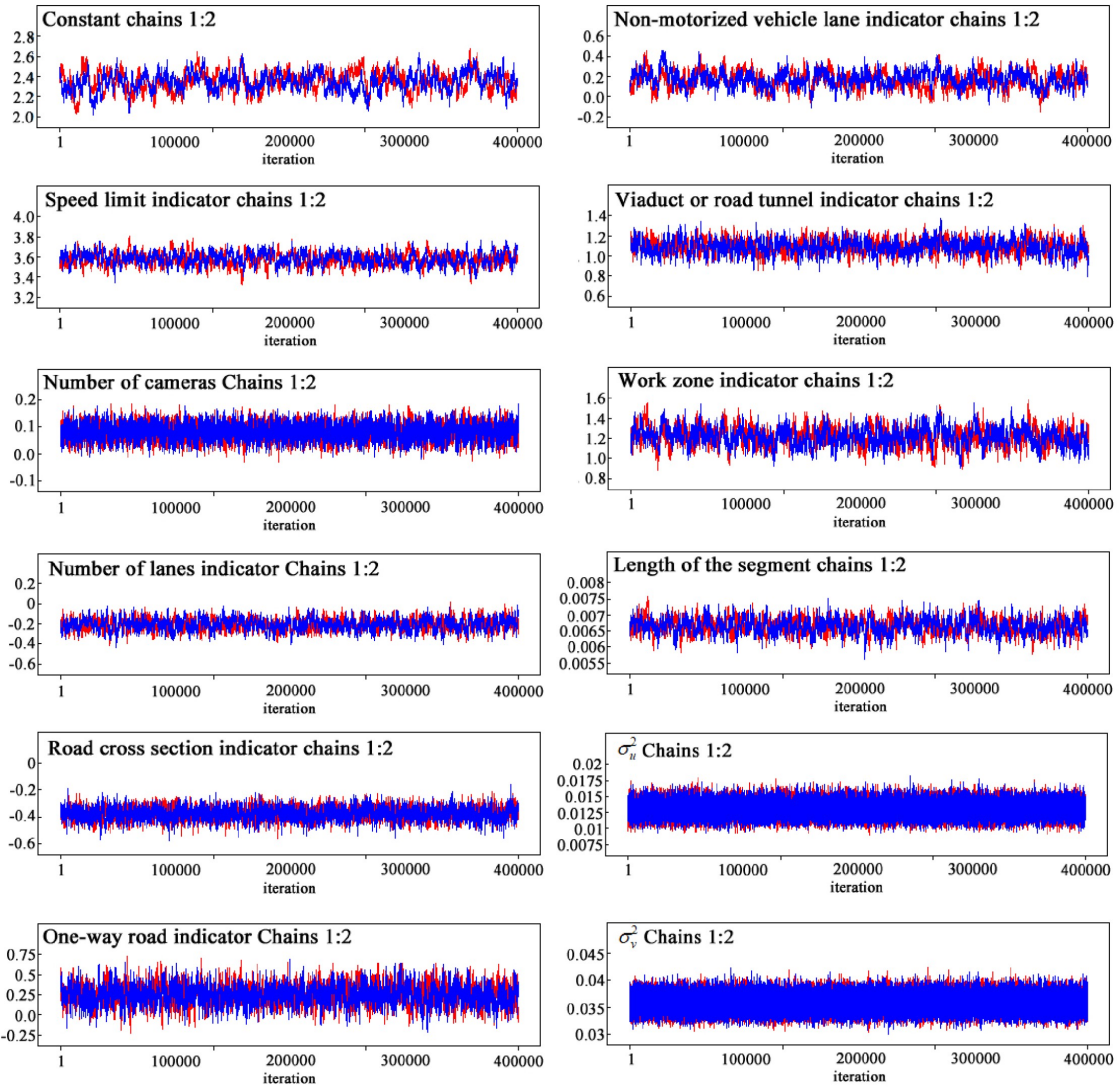
The trace plots for the BHSPLM of serious speeding events.

For the purpose of estimating factors toward frequencies of total speeding events and serious speeding events, BHPLM, BHSPLM, BHNBLM, and BHSNBLM were established by WinBUGS software. The models used two separate MCMC chains for each parameter with different initial values. Each chain was set to run 400,000 iterations and the first 100,000 iterations were discarded as burn-in samples. Posterior estimates were obtained from the remaining 300,000 iterations. The model convergence was examined through computing the Gelman-Rubin statistic as well as visually checking for the trace plots of parameter chains of the two chains [28–30]. For instance, the trace plots of BHSPLM for estimating total speeding events are shown in Fig 1. It illustrates that the estimation results are well converged, since the two chains of each parameter are well mixed despite the different initial values. Furthermore, the Gelman-Rubin statistic value of every parameter is smaller than 1.1, which also shows the significant convergence. Finally, all models in the current study were convergent after adjustments.

### Four Bayesian hierarchical count models of total speeding events

As shown in Table 2, the DIC values are 286148.060, 4314.957, 2945.490, and 4155.468 for the BHPLM, BHNBLM, BHSPLM, and BHSNBLM, respectively. In general, with the impact of spatial effect, both BHSPLM and BHSNBLM have smaller DIC values than those of BHPLM and BHNBLM. The smaller DIC value difference between the models with and without spatial effect is 158.489, which indicates that the models with spatial effect distinctly outperform the models without spatial effect. Moreover, the DIC value difference between BHSPLM and BHSNBLM is 1209.978, suggesting BHSPLM is better than BHSNBLM. Therefore, the BHSPLM is the best fitted model among four Bayesian hierarchical count models in estimating taxi total speeding event.

**Table 2:** DIC values of four models of total speeding events.

Model	*p*_*d*_	$\bar{D}$	*DIC*
BHPLM	11.060	286137.000	286148.060
BHNBLM	25.467	4289.490	4314.957
BHSPLM	241.050	2704.440	2945.490
BHSNBLM	30.868	4124.600	4155.468

The estimation results of four Bayesian hierarchical models are shown in Table 3. The significance level is set as *p* < 0.05. Note that only significant factors are included in the models. According the DIC value, the estimation of BHSPLM is employed to explain the relationship between road factors and total speeding events. The significant factors in BHSPLM include speed limit indicator, number of lanes indicator, road cross section indicator, non-motorized vehicle lane indicator, bus-only lane indicator, viaduct or road tunnel indicator, work zone indicator and length of the segment, which are discussed as follow.

**Table 3:** Estimation results of four Bayesian hierarchical count models of total speeding events.

Variable	BHPLM				BHNBLM				BHSPLM				BHSNBLM			
	Mean	S.d.	95% C.I.		Mean	S.d.	95% C.I.		Mean	S.d.	95% C.I.		Mean	S.d.	95% C.I.	
			Lower	Upper			Lower	Upper			Lower	Upper			Lower	Upper
Speed limit indicator	2.060*	0.005	2.050	2.069	2.173*	0.108	1.961	2.387	2.538*	0.069	2.401	2.673	2.327*	0.120	2.091	2.560
Number of speed cameras	-0.024*	0.002	-0.028	-0.019	--	--	--	--	--	--	--	--	--	--	--	--
Number of lanes indicator	-0.142*	0.004	-0.149	-0.134	--	--	--	--	-0.170*	0.057	-0.280	-0.059	--	--	--	--
Road cross section indicator	-0.251*	0.003	-0.257	-0.245	-0.508*	0.067	-0.639	-0.378	-0.696*	0.038	-0.770	-0.620	-0.612*	0.070	-0.749	-0.476
One-way road indicator	-0.022*	0.006	-0.034	-0.011	--	--	--	--	--	--	--	--	--	--	--	--
Non-motorized vehicle lane indicator	0.601*	0.004	0.593	0.609	--	--	--	--	0.353*	0.075	0.214	0.510	0.421*	0.119	0.182	0.650
Bus-only lane indicator	0.501*	0.006	0.489	0.513	0.602*	0.106	0.396	0.807	0.248*	0.057	0.137	0.360	0.464*	0.110	0.250	0.678
Viaduct or road tunnel indicator	1.008*	0.005	0.999	1.017	0.837*	0.131	0.578	1.091	0.383*	0.070	0.243	0.517	0.599*	0.137	0.329	0.866
Work zone indicator	1.061*	0.005	1.051	1.072	0.737*	0.146	0.448	1.023	1.244*	0.083	1.083	1.410	1.040*	0.167	0.709	1.365
Length of the segment	0.005*	1.5E-5	0.005	0.005	0.007*	4.2E-6	0.006	0.008	0.007*	2.1E-4	0.007	0.008	0.007*	4.5E-6	0.007	0.008
Constant	5.060*	0.008	5.045	5.075	5.126*	0.112	4.908	5.348	4.667*	0.092	4.460	4.840	4.718*	0.166	4.392	5.040
*σ*_*μ*_^2^	--	--	--	--	--	--	--		0.017*	0.001	0.015	0.020	0.002*	3.7E-4	0.002	0.003
*σ*_*v*_^2^	--	--	--	--	--	--	--		0.038*	0.001	0.036	0.041	1.1E-4*	7.5E-5	1.4E-5	3.0E-4

Note: “--” indicates the variable was not included in the model; “*” indicates the variable is significant at 0.05.

Number of lanes indicator and road cross section indicator have significantly negative effect on the taxi total speeding events, revealing that roads with fewer lanes (equal to or less than 4 lanes) and roads without divider are associated with fewer speeding occurrences. It may be interpreted as the fact that roads with these features are tend to be narrower. Because speeding in urban area requires high demand for lane-changing, taxi drivers have much more difficulties committing speeding behavior on narrow roads.

The estimation values show remaining significant road factors positively contribute to the speeding frequency, including speed limit indicator, non-motorized vehicle lane indicator, bus-only lane indicator, viaduct or road tunnel indicator, work zone indicator as well as length of the segment.

For the speed limit, it shows that speed violation is more common on road with low speed limit (40km/h or less). This is similar to the existing finding confirmed by Lai et al [31] and Zhao et al [32]. The first possible reason may be that most experienced drivers (such as taxi drivers) are inclined to drive with a subjective speed, which are impacted by surrounding vehicles and the awareness of environment safety [33]. This subjective speed usually exceeds the speed limits when road speed limits is low, since the risk of being involved in crash on such roads is slighter and accepted by most drivers. The second reason is that, compared to the roads with low speed limits, higher posted speed limits are typically implemented on main roads or express road with higher traffic volume and more police enforcements, making speeding on these roads to be difficult. For the third reason, it is noted that taxi drivers are always over-burdened by the income pressure, pushing them to be more aggressive when driving on roads with low speed limits in order to complete delivering task as soon as possible [7].

As to the existence of non-motorized vehicle lane and bus-only lane, it can be interpreted that isolated road right is significance that stimulates taxi driver to speeding. In populous country like China, the demand of non-motorized vehicle and bus grows as population increase, raising the numbers of non-motorized vehicle and bus in metropolises. However, it also leads to more unexpected lane-changing (usually caused by non-motorized vehicles) or intrusion behaviors (caused by buses) The setting of non-motorized vehicle lane and bus-only lane can decrease those behaviors, reducing their conflicts to ordinary motor vehicles. Thus, populations such as taxi driver are able to speeding frequently under safer traffic condition without mixed traffic volume.

For the impact of viaduct or road tunnel indicator is similar with previous study [34,35], indicating tunnel with low illumination and monotonous environment may encourage drivers to exceed speed limits, the finding can be explained as isolated road right. Road viaduct, which offers drivers express passageways to go across the busy areas, reduces the conflict of non-motorized vehicle and vehicles from opposite direction, and thus, guaranteeing the isolated road right which leads to more speeding violation in practice.

In respect of work zone indicator, the interpretation can be two folded. On the one hand, work zones in Chengdu are majorly prepared for metro constructions, which are usually located on the main roads with 6 or more lanes. In order to ensure the unobstructed traffic, the traffic managers in work zone still retain 2 or more lanes on each direction during construction period, offering an acceptable traffic condition for driving (and for speeding). On the other hand, the speed limit of work zone is temporary and usually much lower than normal roads (e.g., switch from 60 km/h to 30 km/h). Drivers might fail to notice the new speed signs or obey the correct speed limits in the vicinity of the work zone due to subjective speed choice.

As to the length of the segment, it suggests that the drivers (especially for taxi drivers) tend to commit more speed violations on longer roads, since drivers are able to adapt this road condition and therefore committing more speeding by rush or overtake on road with longer length. Meanwhile, longer road segments usually collect more traffic volume (also, more taxis) from the connected branch roads, producing increasing speeding events as well.

In terms of spatial effect, both spatial correlation and heterogeneity are positively related to total speeding events. The spatial fraction is 0.309, which is far less than 1, showing the contribution of spatial heterogeneity outweighs that of spatial correlation effects. Thus, spatial heterogeneity is more critical on determining speeding events. It can be also implied that the taxi speeding is impacted by various geographic discrepancies such as organization, land-use, traffic flow, which usually leads to spatial heterogeneity.

### Four Bayesian hierarchical count models of serious speeding events

The DIC of four models used to estimate serious speeding frequency are shown in Table 4. Values of BHPLM, BHNBLM, BHSPLM and BHSNBLM are 95318.884, 49580.387, 2452.333 and 2571.556, respectively. The results reveal that the introduction of spatial structure improves the fitness of models and suggests that BHSPLM is the best model for serious speeding estimation, followed by the BHSNBLM (the difference of DIC is 119.223). This trend is similar with the results of total speeding event estimation.

**Table 4:** DIC values of four models of serious speeding events.

Model	*P*_*d*_	$\bar{D}$	*DIC*
BHPLM	10.984	95307.900	95318.884
BHNBLM	10.787	49569.600	49580.387
BHSPLM	176.503	2275.830	2452.333
BHSNBLM	169.806	2401.750	2571.556

Table 5 summaries the parameter estimations of serious speeding events using BHPLM, BHSPLM, BHNBLM, and BHSNBLM. The significance is chosen as *p*<0.05. According the result of BHSPLM, the speed limit indicator, number of cameras, one-way road indicator, non-motorized vehicle lane indicator, viaduct or road tunnel indicator, work zone indicator and length of the segment are positively associated with serious speeding frequency, while number of lanes indicator and road cross section indicator are opposite.

**Table 5:** Estimation results of four Bayesian hierarchical count models of serious speeding events.

Variables	BHPLM				BHNBLM				BHSPLM				BHSNBLM			
	Mean	S.d.	95% CI		Mean	S.d.	95% CI		Mean	S.d.	95% CI		Mean	S.d.	95% CI	
			Lower	Upper			Lower	Upper			Lower	Upper			Lower	Upper
Speed limit indicator	3.361*	0.012	3.337	3.385	3.429*	0.014	3.402	3.456	3.577*	0.061	3.458	3.699	3.534*	0.074	3.392	3.683
Number of cameras	-0.034*	0.005	-0.043	-0.024	-0.066*	0.006	-0.079	-0.054	0.081*	0.026	0.030	0.132	0.081*	0.027	0.028	0.134
Number of lanes indicator	-0.168*	0.007	-0.182	-0.154	-0.220*	0.009	-0.237	-0.202	-0.207*	0.056	-0.316	-0.097	-0.182*	0.060	-0.298	-0.064
Road cross section indicator	-0.224*	0.007	-0.237	-0.211	-0.388*	0.009	-0.405	-0.371	-0.377*	0.044	-0.462	-0.291	-0.371*	0.044	-0.457	-0.283
One-way road indicator	0.268*	0.009	0.249	0.286	0.096*	0.013	0.071	0.122	0.250*	0.120	0.014	0.485	0.303*	0.122	0.070	0.549
Non-motorized vehicle lane indicator	0.898*	0.007	0.884	0.911	1.033*	0.009	1.015	1.051	0.168*	0.082	0.008	0.330	0.161*	0.083	0.004	0.329
Bus-only lane indicator	0.284*	0.015	0.255	0.313	0.072*	0.017	0.038	0.106	--	--	--	--	--	--	--	--
Viaduct or road tunnel indicator	1.424*	0.008	1.408	1.440	1.212*	0.011	1.191	1.233	1.092*	0.069	0.957	1.228	1.084*	0.070	0.950	1.220
Work zone indicator	1.233*	0.009	1.215	1.250	1.173*	0.013	1.148	1.199	1.220*	0.090	1.047	1.403	1.179*	0.094	1.000	1.366
Length of the segment	0.005*	3.2E-5	0.005	0.005	0.004*	4.0E-5	0.004	0.004	0.007*	2.4E-4	0.006	0.007	0.007*	2.4E-4	0.006	0.007
Constant	2.378*	0.017	2.344	2.412	2.817*	0.020	2.777	2.856	2.348*	0.094	2.159	2.528	2.416*	0.099	2.217	2.607
*σ*_*μ*_^2^	--	--	--	--	--	--	--	--	0.013*	9.8E-4	0.011	0.015	0.012*	9.4E-4	0.010	0.014
*σ*_*v*_^2^	--	--	--	--	--	--	--	--	0.036*	0.001	0.033	0.038	0.034*	0.001	0.031	0.036

Note: “--” indicates the variable was not included in the model; “*” indicates the variable is significant at 0.05.

For the negative impacts caused by number of lanes indicator and road cross section indicator, the explanation is similar with the analysis of total speeding events, which confirms that wider main roads can offer more suitable traffic condition for taxi driver to accelerate and commit serious speeding.

As to the speed limit indicator, it can be seen that road with lower speed limit has higher serious speeding frequency. Despite the interpretation which are similar with the occurrence of total speeding events, there is another reason that the classification of serious speeding event is strict. Because it is difficult for taxi drivers to exceed the speed limits over 20% on roads with higher speed limits (e.g., 80 km/h) than roads with low speed limits (e.g., 20 km/h), this strict classification may contribute the negative impact to speed violation, leading to the negative trend with speed limits increase. As a result, serious speeding on road with low speed limit tend to be much more common.

There is an interesting finding that number of cameras and one-way road indicator are positively associated with serious speeding, while a previous literature suggests speed camera impose great effect on restricting serious speeding [36]. This may be explained as the weakness of enforcement using fix speed camera [37]. For experienced taxi drivers, they usually avoid punishment by slowing down near the camera and fiercely accelerate after the pass, which is known as "kangaroo effect" [38,39]. However, this phenomenon can be captured by processing GPS trajectory, and thus, identified more practical serious speeding on the downstream area of speed camera. For the one-way roads, because this organization is typically implemented on roads which offer fast and one-direction pass between adjacent arterial roads, drivers therefore can commit excessive speeding without concerning the conflict from opposite lanes. Hence, the one-way roads are linked to more serious speeding events.

As to the impacts of non-motorized vehicle lane indicator, viaduct or road tunnel indicator, work zone indicator and length of the segment, the factors share similarities with those of total speeding events estimations. To be specific, these factors generally offer ideal traffic condition for taxi drivers, making speeding with higher severity to be possible. The ideal conditions can be sorted as isolated road rights (given by non-motorized vehicle lane indicator), low speed limits (given by work zone indicator) and easy to accelerate (given by viaduct or road tunnel indicator and length of the segment).

In respect of spatial effect components, the value of spatial fraction in taxi serious speeding estimation is 0.265 (less than 1). This result suggests that the effect of spatial heterogeneity is greater than that of spatial correlation, which is in accordance with the impact of total speeding events.

## Conclusions

The current study attempts to explore the relationship between taxi speeding frequency and road factors by identifying speeding event based on GPS trajectory data. Both total and serious taxi speeding event are analyzed. To evaluate the factors regarding speeding frequencies, Bayesian hierarchical count models with Poisson and negative binominal distribution are developed (i.e., BHPLM and BHNBLM). Moreover, their corresponding spatial models are established as the comparative models (i.e., BHSPLM and BHSNBLM). The results suggest that BHSPLM is the best model toward both total and speeding events estimation according to the DIC values. Spatial effect, as well as several road factors are associated with significant impact on total and serious speeding events.

In terms of the impact of road factors. It can be concluded that speed limit indicator, number of lanes indicator, road cross section indicator, non-motorized vehicle lane indicator, viaduct or road tunnel indicator, work zone indicator, and length of the segment significantly impact the frequency of total and serious speeding events simultaneously. Roads with fewer lanes and roads without divider show significant negative effects for speeding frequencies, while roads with low speed limit and longer length, and road with special organizations (non-motorized vehicle lane indicator, viaduct and road tunnel, and work zone) are positively associated with speeding behavior. Besides, there are three factors that show different significance between the estimations of total speeding and serious speeding frequency, which are number of speed cameras, one-way road indicator and bus-only lane indicator. The number of speed camera and one-way road are not associated with the total speeding but are linked with serious speeding, whereas bus-only lane indicator shows the opposite significances.

According to the result of models, spatial effect improves the accuracy of parameter estimations. Spatial correlation and spatial heterogeneity effects are significant for total and serious speeding events. The effect of spatial heterogeneity is superior to spatial correlation, showing the fact that geographic discrepancy of factors majorly contributes to the speeding events. Several limitations of the current paper should be presented. First, this study fails to consider some significant factors identified by earlier speeding researches, such as drivers’ information [40], vehicle types [41], and weather [42]. Due to the limitation of GPS trajectory data, the specific information above cannot be obtained directly. The future researches are appreciated by including extra human information, vehicle and environment factors. Second, this paper only discusses the speeding occurred on main roads, while others road types (e.g., branch road, motorway, rural roads) are excluded. The selection of road type limits the extension of characteristic sample, leading to highly unitary data which is not conducive to the universality of the results. The majority of speed limits in this study are 60km/h (about 79.29% among all roads); 89.06% roads have non-motorized vehicle lanes. Third, this paper adopted spatial component as supplement to helpfully correct the relationship between road factors and speeding frequency, however, temporal component is ignored in this study due to the short range of study period. Previous studies have confirmed the importance of panel data analysis (both spatial and temporal effects) in driving researches [43]. Thus, future researches regarding taxi speeding may consider the spatio-temporal effects simultaneously by using more comprehensive data sources.
